# Supplementary material for: Signatures of sex ratio distortion in humans
Source: bioRxiv. 2026 Feb 7:2026.02.04.702084. Preprint. [Version 1] doi: 10.64898/2026.02.04.702084 (PMC12889636; doi:10.64898/2026.02.04.702084)
Supplement: Supplementary file 1 [file NIHPP2026.02.04.702084v1-supplement-1.pdf]

bioRxiv preprint doi: https://doi.org/10.64898/2026.02.04.702084; this version posted February 7, 2026. The copyright holder for this preprint (which was not certified by peer review) is the author/funder, who has granted bioRxiv a license to display the preprint in perpetuity. It is made available under aCC-BY 4.0 International license.

Supplementary Materials

Example Bayesian calculations used by WARP

Assume the allele D is dominant, makes all carriers affected (100% penetrant), and has an allele frequency of 0.01. Here, *M* and *F* refer to affected (i.e., male) and unaffected (i.e., female).

Bayes's theorem applied to probabilities of parental genotypes:

$$P(M)=\frac{P(dd)}{2}+\mathrm{P}(Dd)+P(DD)=\frac{p^2}{2}+2pq+q^2=\frac{0.9801}{2}+0.0198+0.0001=0.50995$$

$$P(dd \vee M)=\frac{P(M \vee dd)P(dd)}{P(M)}=\frac{0.5\cdot 0.9801}{0.50995}=0.9609765\ldots$$

$$P(Dd \vee M)=\frac{P(M \vee Dd)P(Dd)}{P(M)}=\frac{1\cdot 0.0198}{0.50995}=0.38827\ldots$$

$$P(DD \vee M)=\frac{P(M \vee DD)P(DD)}{P(M)}=\frac{1\cdot 0.0001}{0.50995}=0.000196097\ldots$$

$$P(dd \vee F)=\frac{P(F \vee dd)P(dd)}{P(F)}=\frac{0.5\cdot 0.9801}{1-0.50995}=1$$

Thus, the probabilities of genotypes of offspring are as below. Bayes' rule can be further applied to update these probabilities if the sex of the offspring is known. Here, the father's phenotype will be marked fF or fM, and the mother's will be marked mF or mM. Additionally, the event of the two parents having opposite affected statuses, equivalent to $fF \cap mM \cup fM \cap mF$, will be notated as H.

$$P(dd \vee fF \cap mF)=1$$

$$P(DD \vee H)=0$$

$$P(Dd \vee H)=P(DD \vee fM)+\frac{P(Dd \vee fM)}{2}=0.000196+\frac{0.0388}{2}=0.01959$$

$$P(dd \vee H)=1-P(Dd \vee H)=1-0.1959=0.98041$$

bioRxiv preprint doi: https://doi.org/10.64898/2026.02.04.702084; this version posted February 7, 2026. The copyright holder for this preprint (which was not certified by peer review) is the author/funder, who has granted bioRxiv a license to display the preprint in perpetuity. It is made available under aCC-BY 4.0 International license.

$$P(dd \vee fM \cap mM) = P(dd \vee M)^2 + \frac{P(Dd \vee M)^2}{4} + \frac{2\,P(dd \vee M)\,P(Dd \vee M)}{2} = 0.9609^2 + \frac{0.0388^2}{4} + \frac{2 \cdot 0.9609 \cdot 0.0388}{2} = 0.9609\ldots$$

$$P(Dd \vee fM \cap mM) = \frac{2\,P(Dd \vee M)\,P(dd \vee M)}{2} + \frac{P(Dd)^2}{2} + \frac{2\,P(Dd)\,P(DD)}{2} + \frac{2\,P(DD)\,P(dd)}{1} = 0.03842\ldots$$

$$P(DD \vee fM \cap mM) = \frac{P(Dd)^2}{4} + \frac{2\,P(Dd)\,P(DD)}{2} + P(DD)^2 = 0.00038400\ldots$$

Supplementary tables

bioRxiv preprint doi: https://doi.org/10.64898/2026.02.04.702084; this version posted February 7, 2026. The copyright holder for this preprint (which was not certified by peer review) is the author/funder, who has granted bioRxiv a license to display the preprint in perpetuity. It is made available under aCC-BY 4.0 International license.

Supplementary figures

Phenotype	Genotype dd	Dd	DD
Unaffected	1	0	0
**Affected**	**0.9609**	**0.0388**	**0.000196**

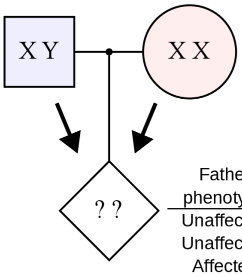

Phenotype	Genotype dd	Dd	DD
**Unaffected**	**1**	**0**	**0**
Affected	0.9609	0.0388	0.000196

Father phenotype	Mother phenotype	Child genotype dd	Dd	DD
Unaffected	Unaffected	1	0	0
Unaffected	Affected	0.98041	0.01959	0
Affected	Unaffected	0.98041	0.01959	0
Affected	Affected	0.9609	0.03842	0.000384

Supplementary Figure 1. ***WARP has high power to infer the likelihood of carrying a distorter in pedigrees regardless of penetrance, inheritance pattern, and other challenges.*** *WARP propagates information about the likelihood of carrying a distorter from parent to child, and from child to parent, using Bayesian network propagation (a combination of Bayes' rule and the chain rule). Here, we depict the probability of two parents transmitting a dominant distorter, D, to a child, assuming that the Dd and DD genotypes cause the affected status 100% of the time, the dd genotype causes the affected status 50% of the time, and the population allele frequency for D is 0.01. The parental genotype likelihoods are calculated using Bayes' rule, and the child's expected genotypes are calculated using the chain rule. See Supplementary Materials for detail. In our study, these assumptions would be used when looking for a Y-chromosome distorter with a 100% distortion ratio and an allele frequency of 0.01.*

bioRxiv preprint doi: https://doi.org/10.64898/2026.02.04.702084; this version posted February 7, 2026. The copyright holder for this preprint (which was not certified by peer review) is the author/funder, who has granted bioRxiv a license to display the preprint in perpetuity. It is made available under aCC-BY 4.0 International license.

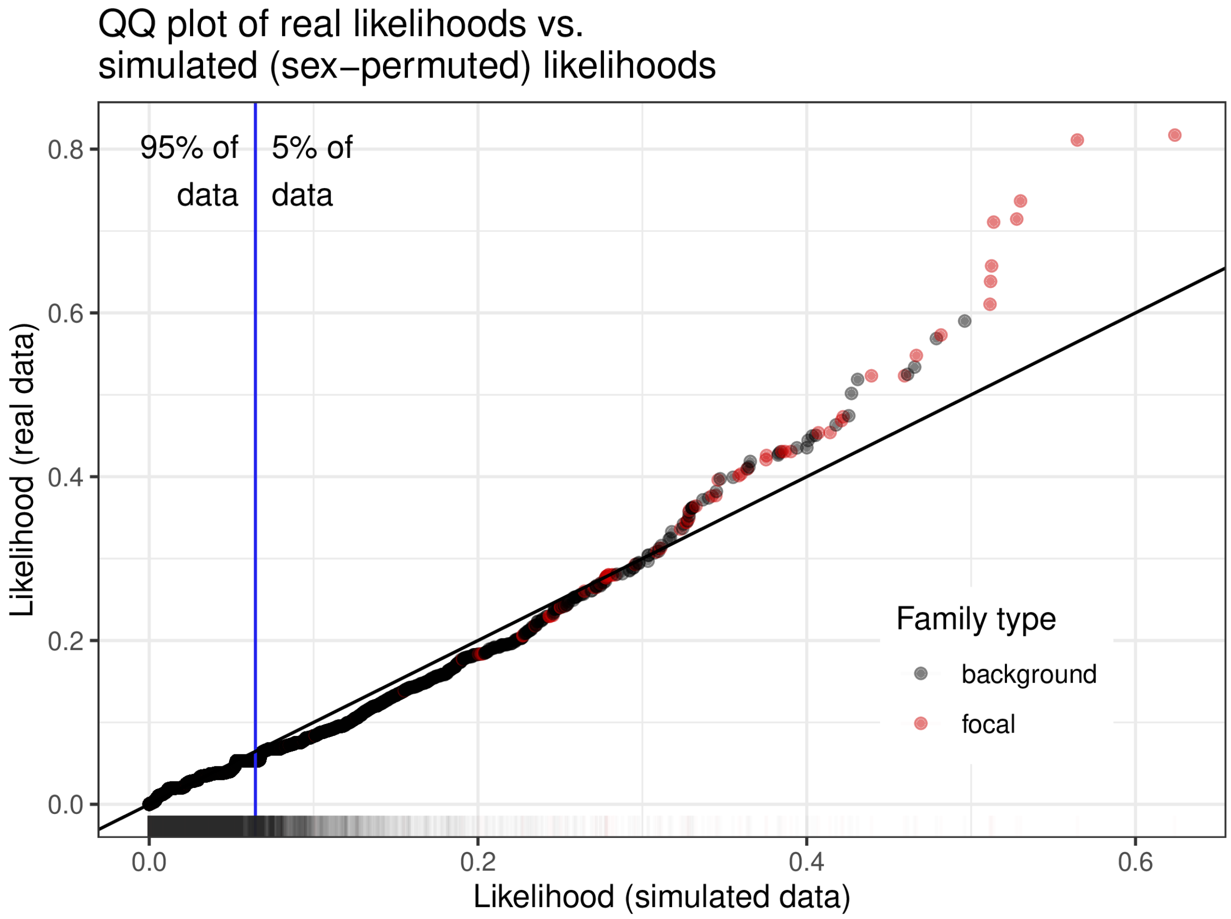

Supplementary Figure 2. ***Likelihoods for highly Y-distorted individuals lie outside the distribution of simulated individuals.*** *This Q-Q plot shows the distribution of Bayesian likelihoods of carrying a Y-biased distorter in the true data (Y axis) and in simulated data (X axis, see Methods for details). The black line is the line of 1:1 correspondence, and the blue line represents the 95th percentile (95% of all data is left of this line). There is a notable deviation away from the 1:1 line in a small number of individuals, most of which are in the putative Y-distorting family (family cluster 2, red).*

bioRxiv preprint doi: https://doi.org/10.64898/2026.02.04.702084; this version posted February 7, 2026. The copyright holder for this preprint (which was not certified by peer review) is the author/funder, who has granted bioRxiv a license to display the preprint in perpetuity. It is made available under aCC-BY 4.0 International license.

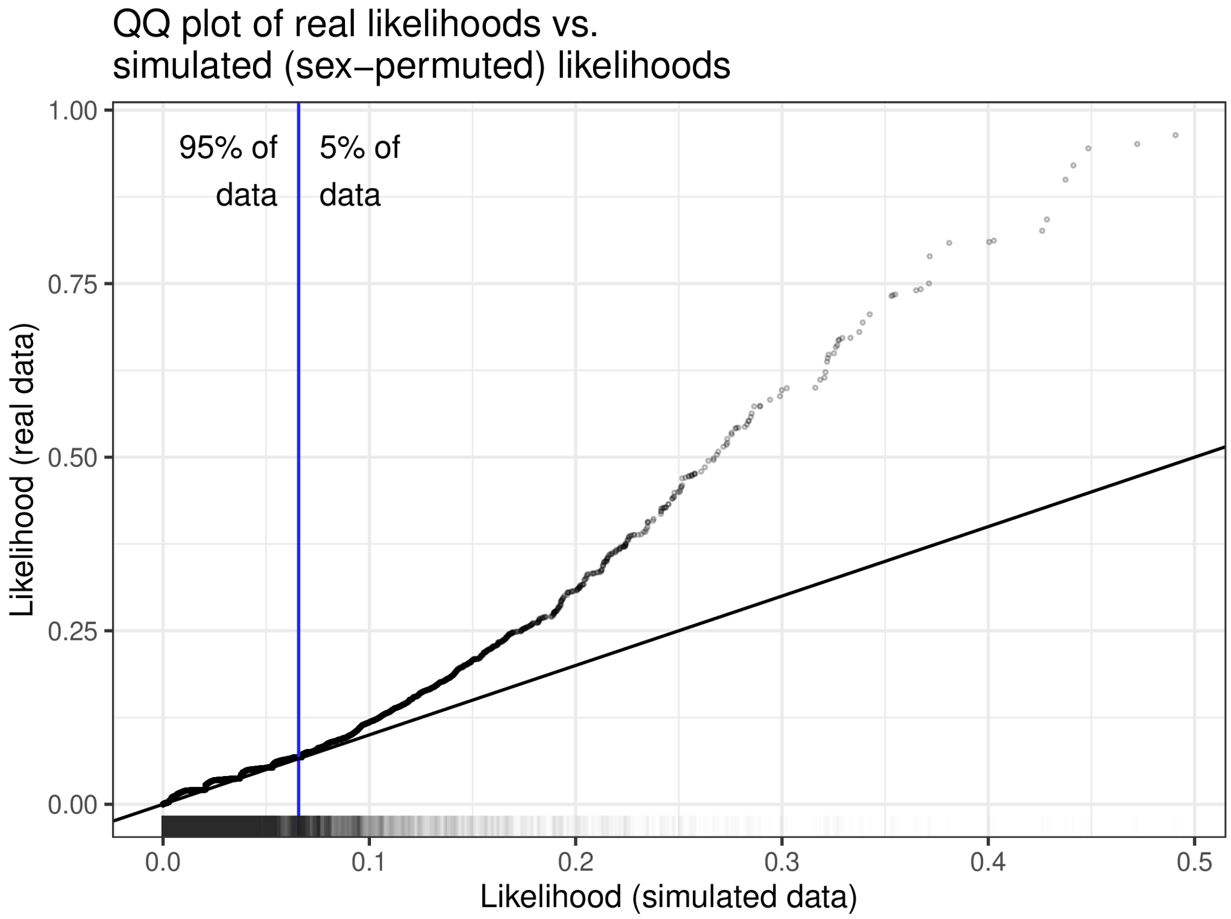

Supplementary figure 3. ***Likelihoods for highly X-distorted individuals lie outside the distribution of simulated individuals.*** *This Q-Q plot shows the distribution of Bayesian likelihoods of carrying an X-biased distorter in the true data (Y axis) and in simulated data (X axis, see Methods for details). The black line is the line of 1:1 correspondence, and the blue line represents the 95th percentile (95% of all data is left of this line). There is a notable deviation away from the 1:1 line in a small number of individuals.*
